# DIAPH3 promotes pancreatic cancer progression by activating selenoprotein TrxR1‐mediated antioxidant effects

**DOI:** 10.1111/jcmm.16196

**Published:** 2020-12-20

**Authors:** Yefei Rong, Jie Gao, Tiantao Kuang, Jianlin Chen, Jian‐ang Li, Yufeng Huang, Haiguang Xin, Yuan Fang, Xu Han, Lun‐Quan Sun, Yue‐Zhen Deng, Zhi Li, Wenhui Lou

**Affiliations:** ^1^ Department of Pancreatic Surgery Zhongshan Hospital Fudan University Shanghai China; ^2^ Xiangya Cancer Center Xiangya Hospital Central South University Changsha China; ^3^ Key Laboratory of Molecular Radiation Oncology Hunan Province Changsha China; ^4^ Department of Oncology Jingjiang Peopleʼs Hospital Affiliated to Yangzhou University Jingjiang China; ^5^ Department of Infectious Disease Ruijin Hospital Shanghai Jiaotong University School of Medicine Shanghai China

**Keywords:** DIAPH3, pancreatic cancer, Selenoamino Acid Metabolism, TrxR1

## Abstract

Pancreatic cancer is a highly malignant tumour of the digestive tract which is difficult to diagnose and treat. Approximately 90% of cases arise from ductal adenocarcinoma of the glandular epithelium. The morbidity and mortality of the disease have increased significantly in recent years. Its 5‐year survival rate is <1% and has one of the worst prognoses amongst malignant tumours. Pancreatic cancer has a low rate of early‐stage diagnosis, high surgical mortality and low cure rate. Selenium compounds produced by selenoamino acid metabolism may promote a large amount of oxidative stress and subsequent unfolded reactions and endoplasmic reticulum stress by consuming the NADPH in cells, and eventually lead to apoptosis, necrosis or necrotic cell death. In this study, we first identified DIAPH3 as a highly expressed protein in the tissues of patients with pancreatic cancer, and confirmed that DIAPH3 promoted the proliferation, anchorage‐independent growth and invasion of pancreatic cancer cells using overexpression and interference experiments. Secondly, bioinformatics data mining showed that the potential proteins interacted with DIAPH3 were involved in selenoamino acid metabolism regulation. Selenium may be incorporated into selenoprotein synthesis such as TrxR1 and GPX4, which direct reduction of hydroperoxides or resist ferroptosis, respectively. Our following validation confirmed that DIAPH3 promoted selenium content and interacted with the selenoprotein RPL6, a ribosome protein subunit involved in selenoamino acid metabolism. In addition, we verified that DIAPH3 could down‐regulate cellular ROS level via up‐regulating TrxR1 expression. Finally, nude mice xenograft model experimental results demonstrate DIAPH3 knock down could decrease tumour growth and TrxR1 expression and ROS levels in vivo. Collectively, our observations indicate DIAPH3 could promote pancreatic cancer progression by activating selenoprotein TrxR1‐mediated antioxidant effects.

## 1 INTRODUCTION

Pancreatic ductal adenocarcinoma (PDAC) is the third most fatal tumour in the United States. It is estimated that the deaths from pancreatic cancer will be second only to lung cancer by 2030.^1^ However, due to lack of effective treatment, the 5‐year survival rate of PDAC patients is still lower than 7%. Gemcitabine is a nucleoside analog that received FDA approval in 1997, and it is still a medicine used in the standard treatment of pancreatic cancer.^2^, ^3^ Adjunctive therapies with Paclitaxel and Erlotinib show a limited curative effect in the treatment of pancreatic cancer.^4^, ^5^ Other treatment options for pancreatic cancer (such as FOLFIRINOX) are highly toxic and only applicable to some patients.^6^ At present, the main genetic driving factors of PDAC have been identified,^7^ of which KRAS is the main driver mutation, and the mutations of several other tumour suppressor genes such as TP53, CDKN2A, SMAD4, BRCA2 and TGFBR2 can promote tumour progression. Unfortunately, as yet there are no targeted medications for these cancer‐driven genes; therefore, it is difficult to design an effective treatment method for PDAC. Only a small proportion of clinically relevant mutations may benefit from existing targeted therapies.^8^

Both mouse and human Formin family proteins contain 15 Formin proteins, characterized by the presence of two formin homology domains. By interacting with the growing ends of actin filaments, Formins can protect it from capping, catalyse the polymerization of actin and regulate the formation of filamentous pseudopods^9^, ^10^, ^11^, ^12^ to support the establishment and maintenance of cell polarity during the development process and in response to disease. Diaphanous formins (including Diaph1, 2 and 3 in mammals) are a subgroup of the Formin family that is homologous to the fruit fly diaphanous gene.^13^ Diaph1‐3 exists in two forms. In an inactivated ‘locked’ form, the carboxy‐terminal diaphanous self‐regulating domain interacts with the upstream inhibitory domain. The binding of the small GTPase to the GTPase‐binding domain disrupts the interaction between the inhibitory domain and the diaphanous self‐regulatory domain, and releases the protein ends, thereby promoting the activation of the Diaph protein.^14^

In Drosophila, mutations in the diaphanous gene cause defects in gamete genesis and neuroblast formation, which are accompanied by defects in cell division caused by polyploids.^15^ In mammals, Diaph1‐3 mutations are associated with local actin cytoskeletal dysfunctions. For example, in Diaph1 and 2 double‐knockout (Diaph1‐2 dko) mice, the radial migration and layer formation of cortical excitatory neurons are basically unaffected, while the tangential migration of cortical interneurons and neuroblasts is impaired from the adult neurogenic subventricular zone (VZ) to the olfactory bulb. F‐actin tissues are destroyed in the migrated interneurons and posterior neuroblasts, thereby weakening the centrosome and nucleus translocation.^16^ Diaph3 is accumulated in the cleavage furrow from the anaphase to the telophase of cell division, and its depletion in the dividing cells in vitro will affect the F‐actin content of the equatorial plate region.^12^, ^17^ In Diaph3‐deficient mice, erythroid cells differentiate normally, but in the telophase of erythroblast cell division, as the actin has not accumulated as much in the cleavage furrow, the progeny cells cannot be separated. Diaph3 overexpressed mice showed internal abnormalities in hair cells. These mice exhibited hearing loss, suggesting that Diaph3 plays an important role in the assembly and/or maintenance of actin filaments in stereocilia. In humans, mutations in the 5'‐untranslated region of the messenger RNA encoding DIAPH3 cause an increase in the expression level of DIAPH3 protein by two to three times compared to normal levels, leading to delayed progressive deafness, which is known as a non‐syndrome dominant chromosome inheritance of auditory neuropathy 1.^18^ In addition, homozygous mutations in the DIAPH3 gene (maternal genetic deletion of 13q and point mutations in the paternal copy) are associated with autism. In addition to its well‐documented role in the actin cytoskeleton, in vitro studies have demonstrated that Diaph3 is associated with the dynamics of microtubules (MTs). Diaph3 is co‐localized with stable MT, and its overexpression is sufficient to generate and target stable MT14. Diaph3 can directly bind (and stabilize) MTs in a way that is independent of actin nucleation.^19^, ^20^ In some literatures, it was reported that Diaph3 interacts with MT tip protein EB1 and colorectal adenomatous polyposis genes (APC) to function as a scaffolding of proteins.^21^

The function of DIAPH3 in tumours differs depending on the tumour type. In prostate and breast cancer cells, DIAPH3 induces nuclear morphological instability and promotes malignant tumour phenotypes.^22^ Consistently, DIAPH3 promotes the growth, migration and metastasis of liver cancer cells by activating the β‐catenin/TCF signalling pathway.^23^ Furthermore, in mouse models, interference with DIAPH3 promotes the invasion and metastasis of tumour cells.^24^, ^25^ The DIAPH3 overexpression inhibits the metastasis and invasive ability of triple‐negative breast cancer by inhibiting the expression of RhoA‐GTP.^26^ However, the specific mechanism of DIAPH3 in the occurrence and development of pancreatic cancer is still unknown.

Excess cytosolic ROS is eliminated by peroxiredoxins using reducing power from thioredoxin reductase‐1 (TrxR1, TXNRD1) or by glutathione peroxidases using reducing power from glutathione reductase (Gsr).^27^ TrxR1 is a key enzyme for redox control of cell function and antioxidant capacity. Many cancer cells have high levels of TrxR1 as a means of surviving their increased endogenous oxidative stress. Thus, TrxR1 is a promising target for improved cancer therapy.^28^

In this study, we investigated the expression pattern, function and molecular mechanism of DIAPH3 in pancreatic cancer, and our observations indicate DIAPH3 could promote pancreatic cancer progression by activating selenoprotein TrxR1‐mediated antioxidant effects.

## 2 MATERIALS AND METHODS

### 2.1 Cell culture

All PDAC cell lines were provided by Professor Yue‐zhen Deng from Xiangya Hospital, Central South University. PDAC cell lines were cultured in DMEM (Gibco) medium containing 10% FBS (Gibco). All cell lines were cultured in a 37℃, 5% CO2 cell incubator.

### 2.2 mRNA extraction and Real‐time PCR

mRNA was extracted using a TRIzol reagent (Invitrogen, 15 596‐026). A reverse transcription kit was used to reversely transcribe cDNAs for Real‐time PCR. The amplification reaction was carried out using a Bio‐Rad system. The reaction system was 20 μl, including10 μl of SYBR Green Mix, 10 μM forward and reverse primers, 1 μl each, 2 μl of template and 6 μl of ribozyme‐free water. The reaction programme was as follows: 95°C for 3 minutes followed by 40 cycles of the reaction, each cycle comprised 95°C for 10s, 60°C for 30s. In order to determine the amplification specificity, the amplification products of each primer pair were subjected to melting curve analysis.

The primer sequences used were as follows:

DIAPH3

Forward：5’‐ GAAACACGGTTGGCAGAGTCT‐3’;

Reverse：5’‐ GTGGCCGTAGTCTCTTCACA‐3’。

β‐actin:

Forward：5’‐ GATCATTGCTCCTCCTGAGC‐3’;

Reverse：5’‐ACTCCTGCTTGCTGATCCAC‐3’。

### 2.3 Whole protein extraction with western blot

After washing with pre‐cooled PBS, cells were lysed with a RIPA lysis buffer (1% NP‐40, 0.1% SDS, 0.5% deoxycholate, 50mM Tris (pH 7.4), Protease Inhibitor Cocktail) for 15 min on ice. After the cells were completely lysed for 15 minutes, the mixture was then centrifuged for 15 min at 4°C, and the supernatant was then transferred. The protein concentration in the lysate was quantitatively analysed with a BCA (Sigma) reagent. The quantified protein was adjusted to the same concentration according to the result of quantitative analysis, then a 4 × loading buffer was added and mixed, and the protein was denatured at 100℃ for 5 min. After it was slightly cooled and centrifuged, the protein was subjected to SDS‐PAGE gel electrophoresis. After electrophoresis, the proteins on the SDS‐PAGE gel were transferred to PVDF membranes (polyvinylidene difluoride membranes, Millipore), and then the target protein bands were cut and blocked at room temperature for 1 hour with TBST containing 5% skim milk powder(25 mM Tris, 150 mM NaCl, 0.05% Tween‐20, pH 7.5), then incubated overnight at 4°C with primary antibodies. The primary antibodies used were DIAPH3 (Abcam), Tubulin (Santa Cruz). The following day, after washing three times with TBST, a secondary antibody was added for incubation for 1 hour at room temperature, and washed three times with TBST, then developed with a millipore luminescent solution.

### 2.4 ICP‐MS analysis

Cells were washed and acid digested. Plates were rapidly washed three times with 100 ml per well of TE buffer (10 mM Tris pH 7.4, 1 mM EDTA). Digestion mix (100 ml per well) was added to all wells and contained 1.5% nitric acid (w/v), 1.5% hydrogen peroxide (w/v) and 50 p.p.b. Ga as an internal standard. Finally, plates were placed in a humidified incubator at 70°C for 2 h. All digested plates were sealed to prevent evaporation and stored at −80°C until ICP‐MS analysis. ICP‐MS analyses were performed at Shanghai USEN Biological Technology Co., Ltd equipped with Agilent Technologies ICP‐MS 7500cs (Santa Clara, CA) and a ESI SC‐4 high‐throughput autosampler.

### 2.5 RNA interference

pLKO.1 lentiviral vector was used to construct shRNA targeting the human DIAPH3 gene in PDAC cells. In order to effectively knock down the expression of DIAPH3 in PDAC cells, we designed and synthesized complementary oligonucleotides, which were annealed and complemented to the linearized pLKO.1 vector. We shared the following two pairs of oligonucleotides for the synthesis of DIAPH3‐shRNA.

sh DIAPH3‐1:

F:

5’‐CCGGCGTGTCAGAATAGCTAAAGAACTCGAGTTCTTTAGCTATTCTGACACGTTTTTTG‐3'

R:

5’‐AATTCAAAAAACGTGTCAGAATAGCTAAAGAACTCGAGTTCTTTAGCTATTCTGACACG3’

sh DIAPH3‐2:

F:

5’‐CCGGGCTCAGTGCTATTCTCTTTAACTCGAGTTAAAGAGAATAGCACTGAGCTTTTTTG‐3'

R:

5’‐AATTCAAAAAAGCTCAGTGCTATTCTCTTTAACTCGAGTTAAAGAGAATAGCACTGAGC‐3’

### 2.6 CCK‐8 proliferation assay

Cells in a logarithmic growth phase were inoculated into a 96‐well plate, 10^3^ cells in each well, and three replicates were established for each group. On the first, third and fifth days, CCK‐8 was added on a 1:10 (by volume) ratio of CCK‐8:culture medium, and incubated at 37℃ for 1 h. The absorbance was measured at a wavelength of 460.

### 2.7 Transwell invasion assay

70 µL of Matrigel diluted with a basal medium (100:3) was added to the centre of the bottom membrane of the upper chamber and incubated at 37℃ for 30 minutes to solidify. A cell suspension was prepared with a culture solution containing 0.1% FBS, and its density was adjusted to 5 × 10^5^ cells/ml. Then, 200 µL of the cell suspension was inoculated into the upper chamber, and 500 µL of a medium containing 10% FBS was added to the 24‐well lower chamber, then placed in an incubator at 37℃ and 5% CO2 for 48 hours, then subsequently fixed with 4% paraformaldehyde and stained with crystal violet. Five fields of view were randomly selected for observation by a 20‐fold microscope, then photographed and counted with Image J software to calculate the relative invasion rate.

### 2.8 Colony formation in soft agar

Double‐layer agar was used in a 24‐well plate, and the lower layer of agar was used to isolate the plate bottom. 1 × 10^3^ cells were mixed with the upper layer of agar and then inoculated into the 24‐well plate. Two weeks later, the non‐anchored growth condition of cells was observed and photographed, and then statistical analysis was performed.

### 2.9 Silver staining

A Beyotime silver staining kit was used for silver‐staining analysis.

### 2.10 Bioinformatics analysis

The DIAPH3 interacting protein network diagram was drawn with cytoscape software. The GO analysis of the DIAPH interacting protein was detected with mass spectrometry and the network construction of the interacting proteins was completed using the Metascape website (http://metascape.org/gp/index.html#/main/step1) and visualized with Cytoscape.

### 2.11 Measurement of ROS

Intracellular accumulation of ROS was determined using the fluorescent probes 2’, 7’‐dichlorodihydrofluorescein diacetate (H2DCFDA). Miapaca‐2 and CFPAC‐1 cells were pretreated with 20 μM H2O2 for 30 minutes. Cells were then stained with the fluorescent dye DCFDA for an additional 10 minutes. Then, cells were washed in PBS twice, and fluorescence was detected by fluorescence microscope. Meanwhile, the ROS levels were measured by flow cytometry and analysed using the FlowJo software.

### 2.12 Mouse models

All the experimentation for animals was approved by Animal Ethics Committee of Fu Dan University, following the Guidelines of Animal Handling and Care in Medical Research from Shang Hai, China. For human PDAC xenograft model, 6–8 weeks old male nude mice were from Hunan SJA Laboratory animal company. Mia Paca‐2 cells (1.7 × 106) or CFPAC‐1 cells (3.0 × 106) virally transformed with control vector or shDIAPH3 were injected into the left (shDIAPH3) and right (control vector) flank of nude mice for PDAC tumour formation, respectively.

### 2.13 IHC staining assay

The samples of PDAC tissue arrays or syngeneic tumour were subjected to heat‐mediated antigen retrieval in 0.01 mol/l citrate buffer (pH 6.0). After cooling to room temperature, samples were treated successively with 1% methanol/30% H_2_O_2_ to block endogenous peroxidase and 5% bovine serum albumin to block nonspecific binding sites. After rinsing with PBS, they were incubated overnight at 4°C with the Rabbit‐anti‐DIAPH3 or Rabbit‐anti‐TrxR1 primary antibody. The sections were then rinsed with PBS and incubated with the HRP conjugated secondary antibody for 30 min at room temperature. Then the sections were washed and incubated with DAB (Abcam, Cambridge, UK) and were terminated by rinsing with distilled H_2_O. Finally, the samples were counterstained with haematoxylin.

## 3 RESULTS

### 3.1 DIAPH3 Expression was up‐regulated in Pancreatic Cancer

**FIGURE 1:**
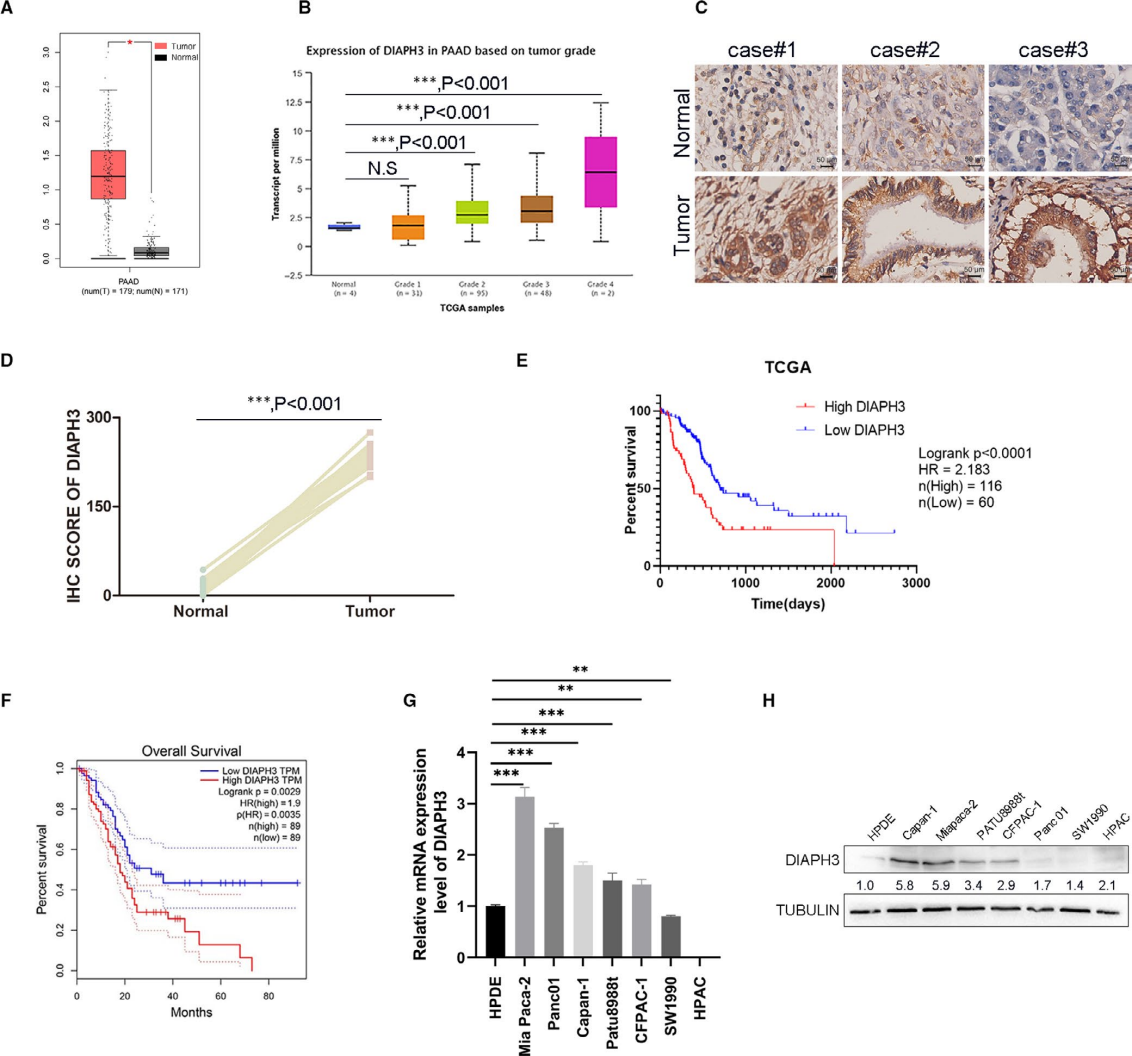
The expression of DIAPH3 was up‐regulated in pancreatic cancer (A) The mRNA expression level of DIAPH3 in patients with pancreatic cancer in the GEPIA database. Left (red): tumour (N = 179); right (grey): normal (N = 171), *P* < 0.05, error bars denote mean ± SEM. (B) mRNA expression levels ofDIAPH3 in different grades of pancreatic cancer in TCGA samples of pancreatic cancer in the UALCAN database, error bars denote mean ± SEM, ***P < 0.001; N.S, no significant. (C) Immunohistochemical analyses of DIAPH3 in patients’ pancreatic tissue. (D) Quantification of IHC score in (C), (five fields each; mean ± SEM.; paired *t* test). (E) Survival analysis based on the level of DIAPH3 expression in pancreatic cancer patients in TCGA database. (F) Survival analysis based on the level of DIAPH3 expression in pancreatic cancer patients in the GEPIA database. *P < 0.05, ***P < 0.0001, Mann‐Whitney test. All experiments were repeated three times. (G) mRNA expression levels of DIAPH3 in pancreatic cancer cell lines, Error bars denote mean ± SEM. (H) Expression of DIAPH3 protein levels in pancreatic cancer cell lines (normal pancreatic ductal cells: HPDE; PDAC cells: Capan‐1, Miapaca‐2, PATU8988t, CFPAC‐1, Panc01, SW1990, HPAC)

Pancreatic cancer has a high malignancy and poor survival prognosis. Therefore, the study on the effect of cancer‐promoting genes on the occurrence and development of pancreatic cancer and further understanding of its mechanism can provide better basis for the treatment of pancreatic cancer. According to the database GEPIA (Gene Expression Profiling Interactive Analysis), we found that the DIAPH3 expression was significantly up‐regulated in pancreatic cancer, and the expression level of DIAPH3 in tumour tissues of patients with pancreatic cancer was 6–7 times that of normal tissue (Figure 1A). We further analysed the data in the TCGA database UALCAN and confirmed the up‐regulated expression of DIAPH3 in pancreatic cancer, and this up‐regulation multiple gradually increased along with the progression of pancreatic cancer (Figure 1B). In support of this data‐mining result, we verified DIAPH3 was overexpressed in pancreatic tumour tissues over adjacent tissues with paired clinical samples (Figure 1C‐D). Further analysis showed that patients with high expressions of DIAPH3 in pancreatic cancer had a shorter survival than patients with low expressions (Figure 1E and Figure 1F). qPCR result showed that the transcription level of DIAPH3 in several pancreatic cancer cells was higher than that of normal pancreatic cells HPDE (Figure 1G), and a similar conclusion was obtained at the protein level (Figure 1H). Overall, the expression of DIAPH3 is up‐regulated in pancreatic cancer and may be closely associated with the occurrence and development of pancreatic cancer.

### 3.2 Proliferation of pancreatic cancer cells was promoted by DIAPH3

**FIGURE 2:**
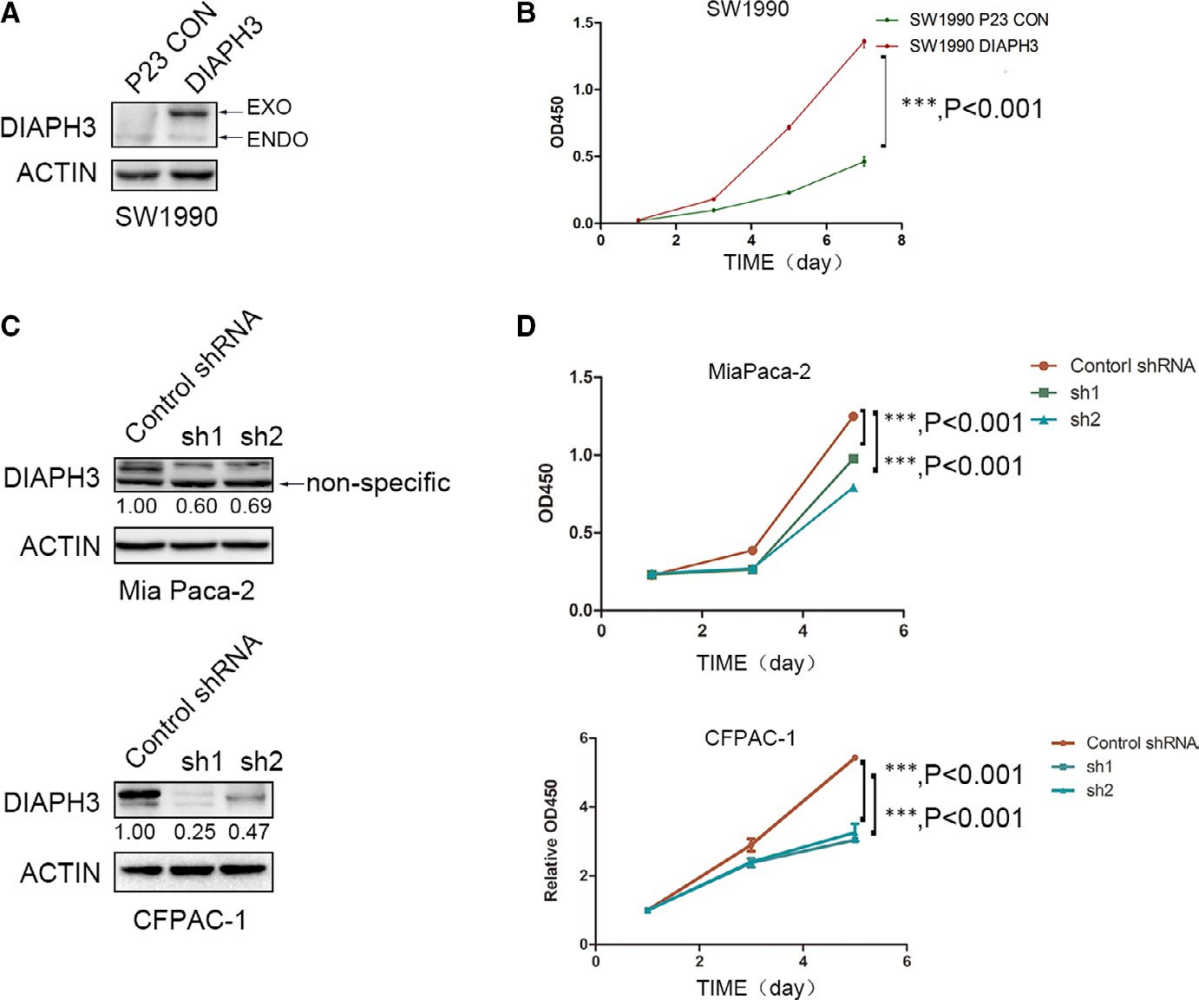
DIAPH3 promotes the proliferation of pancreatic cancer cells. (A) The overexpression efficiency of DIAPH3 in pancreatic cancer cells SW1990 was detected using Western Blot. (B) Detection of the changes in the proliferation rate of pancreatic cancer cells SW1990 after overexpression of DIAPH3 with a CCK‐8 assay. Error bars denote mean ± SEM. (C) detection of the knock down efficiency of DIAPH3 in pancreatic cancer cells (Mia Paca‐2, CFPAC‐1) using Western Blot. (D) Detection of the changes in the proliferation ability of pancreatic cancer cells Mia Paca‐2 and CFPAC‐1 after DIAPH3 knock down using CCK‐8 assay, error bars denote mean ± SEM *P* < 0.0001, Mann‐Whitney test. All experiments were repeated three times

In order to further study the function of DIAPH3 in pancreatic cancer cells, we established a stable overexpressing cell line of DIAPH3 in pancreatic cancer cells SW1990 and Miapaca‐2 (hereinafter referred to as Mia Paca‐2) (Figure 2A and Figure S1A). The cell proliferation rate was measured with a CCK8 assay, and it was found that the proliferation rate of pancreatic cancer cells overexpressing DIAPH3 was significantly higher than that of the control cells. The same results were observed in both SW1990 and Mia Paca‐2 pancreatic cancer cells (Figure 2B and Figure S1B). In order to further verify the functions of DIAPH3 in promoting the proliferation of pancreatic cancer, we designed an shRNA targeted to human DIAPH3, constructed DIAPH3 stable knock down cell line in Mia Paca‐2 and CFPAC‐1 cells and tested its knock down efficiency using Western Blot (Figure 2C). Compared with control cells, both shRNAs can effectively knock down DIAPH3 in different cell lines. After knocking down DIAPH3 in pancreatic cancer cells, its proliferation rate was significantly inhibited (Figure 2D). We then constructed another two DIAPH3 knock down cell lines in SW1990 and HPDE. In accordance with these two knock down cell lines results, knock down of DIAPH3 significantly decreased proliferation capacity of SW1990 and HPDE cells (Figure S1C‐F). Collectively, we regulated the expression level of DIAPH3 in pancreatic cancer cells and found that DIAPH3 could indeed promote the proliferation of pancreatic cancer cells.

### 3.3 DIAPH3 promotes pancreatic cancer cell invasion

**FIGURE 3:**
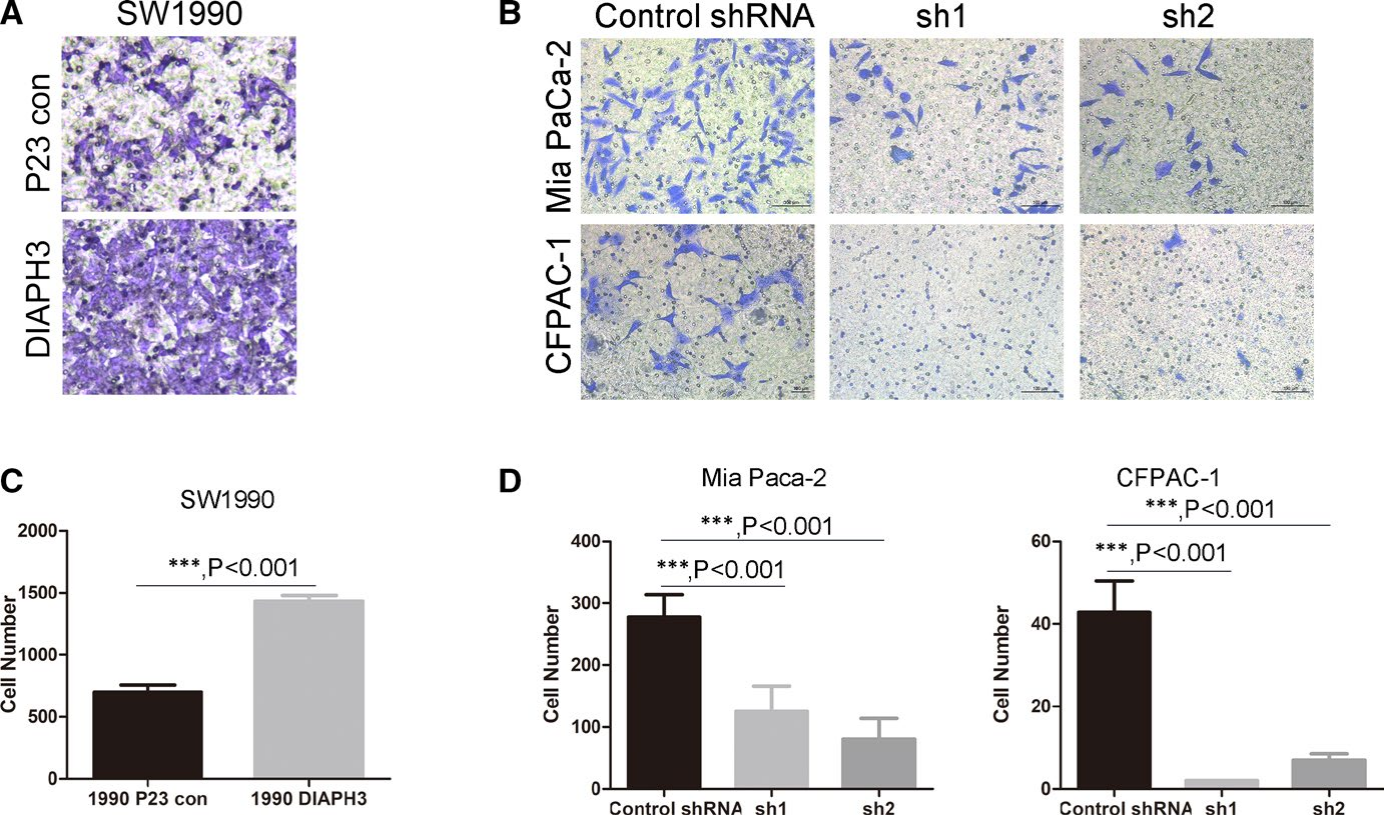
DIAPH3 promotes the invasion of pancreatic cancer cells. (A and C) (A) Detection of the invasive efficiency of DIAPH3 overexpressing cell line SW1990 established in FIG2A using a Transwell assay and statistical analysis was presented in (C), Error bars denote mean ± SEM. (B and D) Detection of the invasive capacity of DIAPH3 knock down cell line of pancreatic cancer cells Mia Paca‐2 and CFPAC‐1 established in FIG2C using a Transwell assay (B) and statistical analysis (D) Error bars denote mean ± SEM. ***P < 0.0001, Mann‐Whitney test. All experiments were repeated three times

Pancreatic cancer has an extremely low survival rate because of its high degree of malignancy and the subsequent multiple metastases during the early stages. Therefore, we should study the metastasis of pancreatic cancer in addition to its occurrence and development. Given that we have discovered the regulatory function of DIAPH3 on the proliferation of pancreatic cancer cells (Figure 2), we further investigated the effect of DIAPH3 on the invasive capacity of pancreatic cancer cells. We used the cell lines established in pancreatic cancer cells to conduct our study and found that DIAPH3 could regulate the invasive capacity of pancreatic cancer cells. The cell invasive capacity was detected using a Transwell assay. After overexpressing DIAPH3 in pancreatic cancer cells, its invasive capacity was greatly enhanced, which was verified in SW1990 cell lines (Figure 3A and C), while DIAPH3 did not affect cell proliferation under this low serum culture concentration (0.1%) within 48 hours (Figure S2A), which rules out the possibility of false‐positive observation of DIAPH3 enhanced invasion resulted from DIAPH3‐induced proliferation. In order to further confirm this function, we also used pancreatic cancer cell lines Mia Paca‐2 and CFPAC‐1 with DIAPH3 knock down to conduct a study and found that the invasive capacity of pancreatic cancer cells was greatly weakened after knock down of DIAPH3 (Figure 3B and D), likewise, DIAPH3 did not have an impact on cell proliferation of Mia Paca‐2 and CFPAC‐1 under this low serum culture concentration (0.1%) within 48 hours (Figure S2B and C). In summary, DIAPH3 can promote the invasive capacity of pancreatic cancer cells.

### 3.4 DIAPH3 promotes tumour formation/non‐anchored growth of pancreatic cancer cells

**FIGURE 4:**
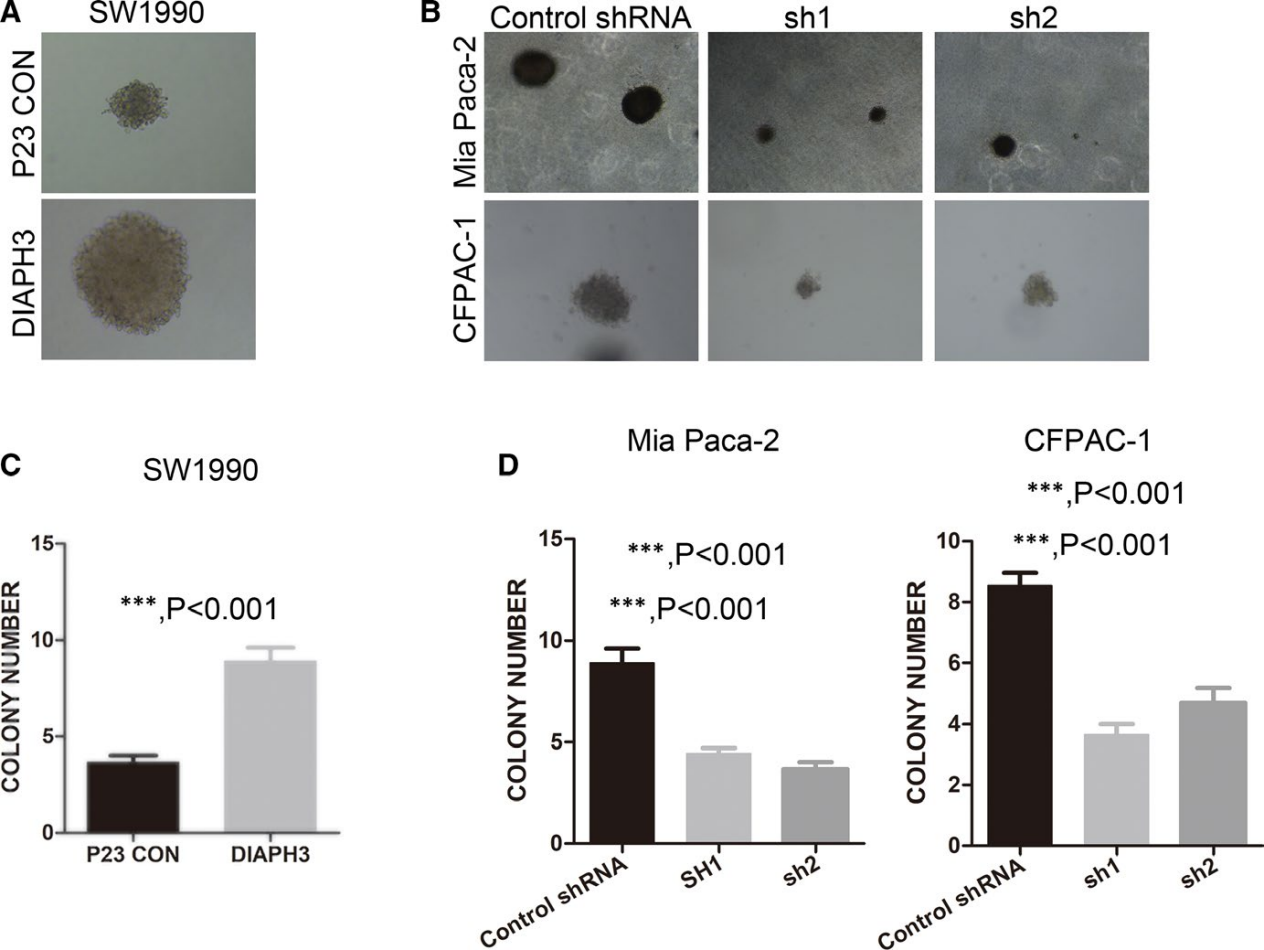
DIAPH3 promotes tumour formation and non‐anchored growth ability of pancreatic cancer cells. (A and C) (A) Non‐anchored growth ability of DIAPH3 overexpressed cell lines SW1990 was investigated by soft agar assay and statistical analysis was presented in (C), Error bars denote mean ± SEM. (B and D) Non‐anchored growth ability of DIAPH3 knock down PDAC cell lines Mia Paca‐2 and CFPAC‐1 established in Figure 2C was investigated using soft agar assay, and statistical analysis was presented in (D), Error bars denote mean ± SEM. ***P < 0.0001, Mann‐Whitney test. All experiments were repeated three times

After confirming the effect of DIAPH3 on the occurrence and metastasis of pancreatic cancer, we further confirmed its effect on the development of pancreatic cancer. When overexpressing DIAPH3, the non‐anchored growth ability of pancreatic cancer cells SW1990 was significantly up‐regulated (Figure 4A and C). Similarly, when we knocked down DIAPH3 in pancreatic cancer cells Mia Paca‐2 and CFPAC‐1, the non‐anchored growing ability of pancreatic cancer cells was significantly blocked (Figure 4B and D). Therefore, it is possible to inhibit the progression of pancreatic cancer through DIAPH3 interference.

### 3.5 bioinformatic analysis shows DIAPH3 may promote pancreatic cancer progression via regulation of selenoamino acid metabolism

**FIGURE 5:**
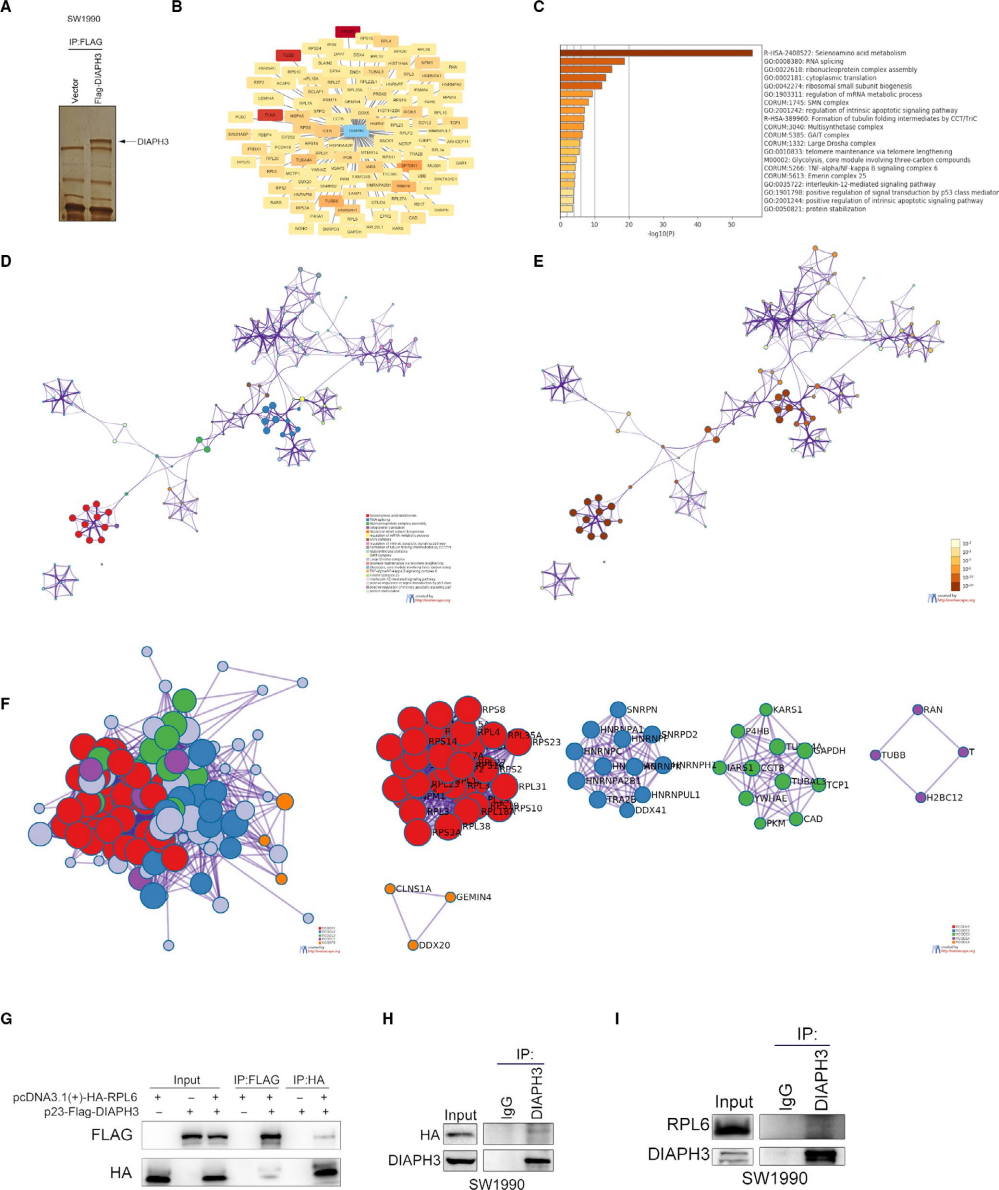
Identification and bioinformatic analysis of candidate proteins interacted with DIAPH3. (A) The silver‐staining diagram of DIAPH3‐interacted proteins with CO‐IP assay. (B) Proteins interacted with DIAPH3 identified with Mass spectrometry were presented using Cytoscape software. The colour of each node was denoted according to the value of PSM (Peptide‐Spectrum Match). (C) GO analysis of DIAPH3 interacting proteins, sorted according to the p value. (D‐E‐H) Network display of DIAPH3 interacting protein. Network nodes represented terms analysed by GO. (D) Similar terms in GO classification were given the same colour. (E) Terms with similar P values in the GO classification were given the same colour. (F) By extracting the interaction relationship between the DIAPH3 interacting proteins in the BioGrid, InWeb_IM and OmniPath databases, the interacting proteins were divided into 5 MCODE subnetworks according to the closeness of the interaction relationship using the Molecular Complex Detection (MCODE) algorithm, and the final graphic display was performed by cytoscape software. (G) Validation of the exogenous interaction of DIAPH3 with RPL6 using CO‐IP assay. (H) Detecting the interaction of endogenous DIAPH3 with exogenous HA‐RPL6 in pancreatic cancer cell (SW1990) by CO‐IP assay. (I) Verifying the endogenous interaction of DIAPH3 with RPL6 in pancreatic cancer cell (SW1990) by CO‐IP assay

In order to study the molecular mechanism of DIAPH3 in promoting the progression of pancreatic cancer, we screened DIAPH3 interaction proteins by mass spectrometry (Figure 5A). A total of 116 DIAPH3‐specific interacting proteins were identified by mass spectrometry (Table S1), among which RPS8, IARS, RPL4, RPL6 and RPS2 are top 5 most identified proteins. Through GO analysis on the Metascape website, we found that the interacting proteins of DIAPH3 were mainly selenoamino acid metabolic enzymes (Figure 5B, C, E, F), indicating that DIAPH3 may affect the metabolism of selenoamino acids in pancreatic cancer. We further investigated the GO analysis results and performed similarity correlation of significantly enriched GO terms in the interacting proteins, and found that genes of the selenoamino acid metabolic pathways were independent of the GO terms of other enrichment analysis compared with other DIAPH3 interacting proteins with a smaller P value; therefore, it showed an extremely significant enrichment (Figure 5D, E, G, H). Further, we performed a networking display of the proteins obtained by GO enrichment analysis by using the protein interaction relationships in the three protein interaction databases BioGrid, InWeb_IM and OmniPath, and found that the genes of the selenoamino acid metabolic pathways that interact with NIDPH3, such as RPL23, RPL31, RPL14 could also interact with each other, and the Molecular Complex Detection (MCODE) algorithm indicated that most genes of the selenoamino acid metabolic pathway were aggregated into subnetwork 1 (Figure 5F, MCODE_1). Therefore, we speculated that DIAPH3 may interact with key genes of various selenoamino acid metabolic pathways in pancreatic cancer to significantly impact the functions of genes related to selenoamino acid metabolic pathways, and promote selenoprotein (such as TrxR1 or GPX4) synthesis, which direct reduction of hydroperoxides or resist ferroptosis, respectively. Moreover, we conducted CO‐IP assay and validated the interaction of exogenous DIAPH3 with exogenous RPL6, and endogenous DIAPH3 with exogenous/endogenous RPL6 (Figure 5G, H and I) in PDAC cell. However, we failed to validate the potential interaction between DIAPH3 and any protein of RPS8, IARS, RPL4 and RPS2 with Co‐IP (data not shown).Thus, DIAPH3 may be involved in selenium metabolism of PDAC cells via interaction with RPL6.

### 3.6 DIAPH3 promotes malignant phenotype of PDAC cells via promoting selenoprotein TrxR1‐mediated antioxidant effects

**FIGURE 6:**
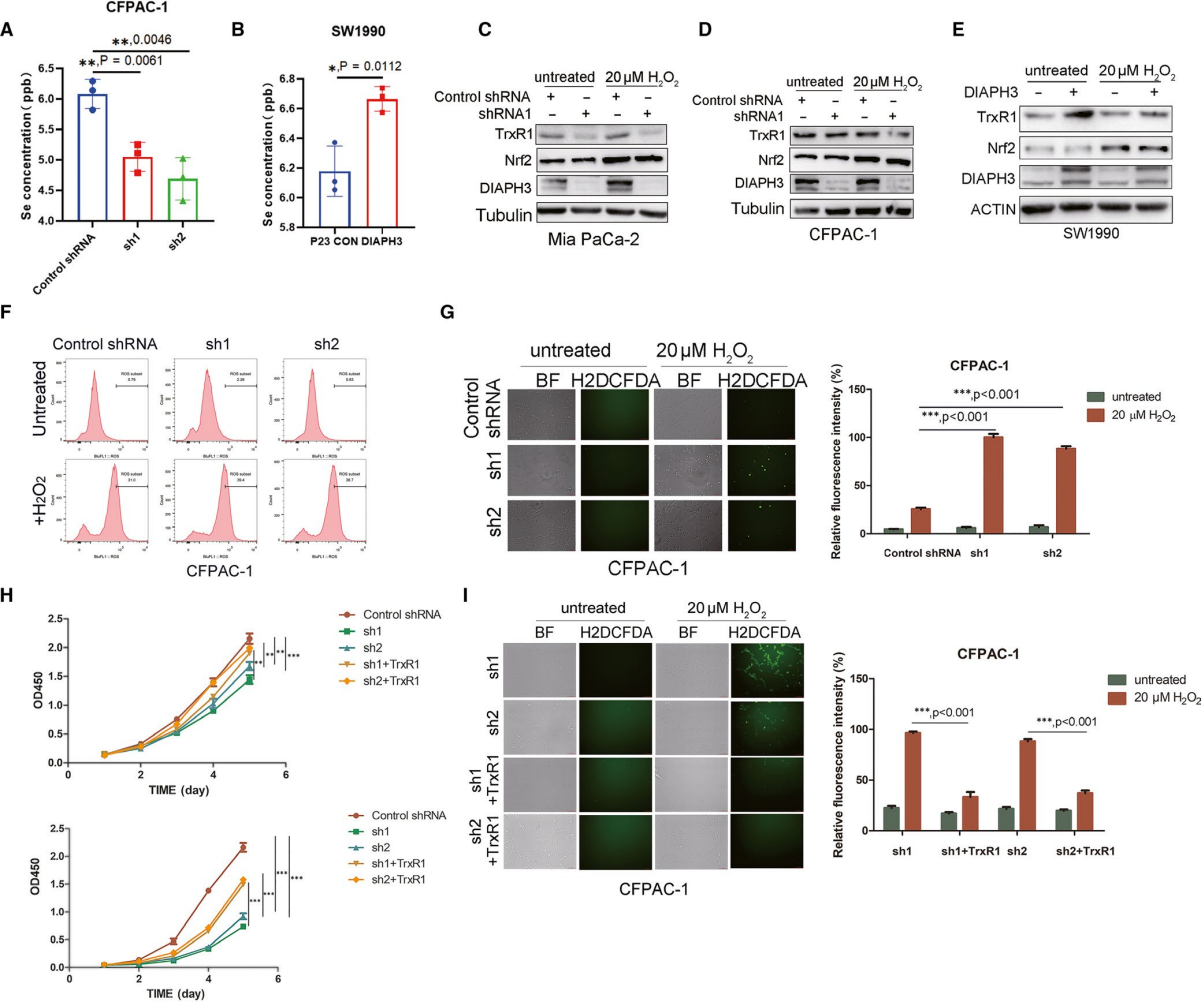
DIAPH3 Promotes PDAC Cells Malignant Phenotype via TrxR1‐Mediated ROS Levels. (A) Detection of selenium concentration of DIAPH3 knock down PDAC cell line CFPAC‐1 using ICP‐MS (Inductively coupled plasma mass spectrometry). (B) Selenium concentration of DIAPH3 overexpressing cell line SW1990 measured by ICP‐MS (Inductively coupled plasma mass spectrometry). (C and D) Control and DIAPH3 knock down pancreatic cancer cells Mia Paca‐2 (C) and CFPAC‐1 (D) were treated with or without 20 μM H_2_0_2_, after which the expression of TrxR1 and DIAPH3 was detected with Western blot. (E) Control and DIAPH3 overexpressed PDAC cell SW1990 were treated with or without 20 μM H_2_0_2_, after which the expression of TrxR1 and DIAPH3 were detected with Western blot. (F) Detection of the ROS level of DIAPH3 knock down cell line CFPAC‐1 treated with or without 20 μM H_2_0_2_ using flow cytometry after stained with H2DCFDA. (G) Control and DIAPH3 knock down CFPAC‐1 cell lines were treated with or without 20 μM H H_2_O_2_, after which ROS level of these cells was detected with immunofluorescence microscope after stained with H2DCFDA. (H) CCK‐8 assay was performed on control and TrxR1 overexpressing PDAC Mia Paca‐2 and CFPAC‐1 cells after DIAPH3 knock down. (I) ROS levels of control and TrxR1 overexpressing PDAC CFPAC‐1 cells after DIAPH3 knock down were detected with H2DCFDA after treatment with or without 20 μM H_2_O_2_

In order to investigate the accurate molecular mechanism underlying regulation of selenoamino acid metabolism by DIAPH3, we first explored the potential regulation of selenium metabolism and selenoprotein TrxR1 and GPX4 by DIAPH3. Interestingly, we verified knock down or overexpression of DIAPH3 could significantly decrease or increase selenium levels in PDAC cells (Figure 6A and B). In addition, DIAPH3 knock down could significantly decrease TrxR1 expression in Mia Paca‐2 and CFPAC‐1 cells (Figure 6C and D, Figure S3B) and overexpression of DIAPH3 could increase the expression of TrxR1 in SW1990 cells (Figure 6E). However, GPX4 expression was not impacted by overexpression or knock down of DIAPH3 (Figure S3A). Thus, DIAPH3 may promote TrxR1 expression via increasing selenium levels in PDAC cells. TrxR1 belongs to the pyridine nucleotide‐disulphide oxidoreductase family, and is a member of the thioredoxin (Trx) system. TrxR1 reduce thioredoxins as well as other substrates and play a key role in redox homoeostasis via decreasing the concentrations of reactive oxygen species (ROS). Thus, we explored the impact of DIAPH3 on ROS levels of PDAC cells. As expected, knock down of DIAPH3 significantly increases cellular ROS levels (Figure 6F,G, Figure S3D and E), while DIAPH3 overexpression decrease ROS levels (Figure S3C). Thus, DIAPH3 could regulate ROS level of PDAC cells. Next, we aimed to investigate the implication of TrxR1 in regulation of ROS levels by DIAPH3. We overexpressed TrxR1 in DIAPH3 knock down Mia Paca‐2 and CFPAC‐1 cells and verified the expression of TrxR1 (Figure S3F). As speculated, overexpression of TrxR1 alleviated increase of ROS levels upon treatment with H_2_O_2_ in DIAPH3 knock down cells (Figure 6I, Figure S3G). More importantly, TrxR1 overexpression rescued the proliferation inhibition phenotype imposed by DIAPH3 knock down in Mia Paca‐2 and CFPAC‐1 cells(Figure 6H). Collectively, these data indicate DIAPH3 could promote PDAC cells malignant phenotype via promoting selenoprotein TrxR1‐mediated antioxidant effects.

### 3.7 DIAPH3 knock down could decrease tumour growth and TrxR1 expression in vivo

**FIGURE 7:**
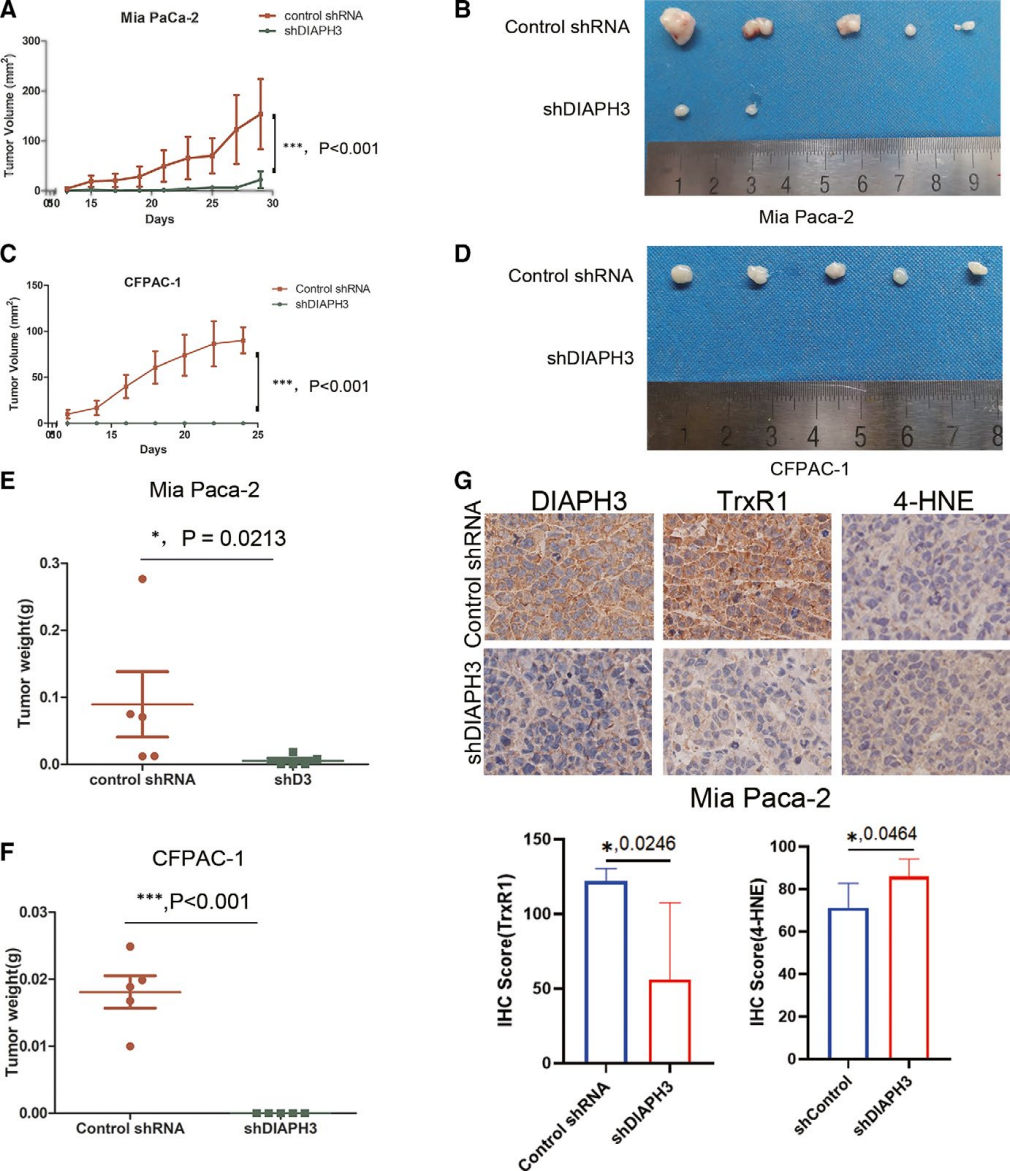
DIAPH3 Knock down Could Decrease Tumour Growth and TrxR1 Expression in vivo. (A) Tumour growth curve of DIAPH3 knock down and control Mia Paca‐2 cells in nude mice xenograft model. Each point represents the mean tumour volumes of five mice; bars represent the SD. (***P < 0.001, Student's *t* test). (B) Tumour xenografts appearance of nude mice inoculated with Mia Paca‐2 cells for 29 days. (C) Quantification of tumour xenografts weight in (B). Each point represents the mean tumour weight of five mice; bars represent the SD. (***P < 0.001, paired *t* test). (D) Tumour growth curve of DIAPH3 knock down and control CFPAC‐1 cells in nude mice xenograft model. Each point represents the mean tumour volumes of five mice; bars represent the SD. (***P < 0.001, Student's *t* test). (E) Tumour xenografts appearance of nude mice inoculated with CFPAC1 cells for 24 days. (F) Quantification of tumour xenografts weight in (D). Each point represents the mean tumour weight of five mice; bars represent the SD. (***P < 0.001, paired *t* test). (G) IHC staining of DIAPH3 , TrxR1 and 4‐HNE of PDAC xenografts of inoculated nude mice taken from (B) (*P < 0.05, Student's *t* test)

Finally, in order to verify whether our discoveries were applicable in vivo, we inoculated control and DIAPH3 knock down Mia Paca‐2 and CFPAC‐1 cells into nude mice and investigated the impact of DIAPH3 on xenograft growth. In accordance with our in vitro data, DIAPH3 knock down significantly alleviated tumour growth of Mia Paca‐2 cells (Figure 7A, B and E) and even prevents tumour of CFPAC‐1 cells from growing out (Figure 7C, D and F). In addition, IHC assay demonstrated DIAPH3 knock down significantly decreased TrxR1 expression, while expression of 4‐HNE (a marker of lipid peroxidation and ROS in IHC assay^29^) was increased in vivo(Figure 7G). Taken together, our observations indicate DIAPH3 knock down could decrease tumour growth and TrxR1 expression in vivo.

## 4 DISCUSSION

PDAC is a ductal adenocarcinoma that originates in the epithelium of the glandular duct, and its morbidity accounts for 85% of all pancreatic cancers^30^ and has the poorest prognosis. Although better animal models have been established over the past few decades and humans have made great progress in understanding the biology of PDAC, there is still no effective means of treatment. Among patients with PDAC, about 15% to 20% of patients meet the indications for surgery, which is a small proportion, and more than 80% of patients relapse after undergoing surgical resection. At present, various types of chemotherapeutic drugs for PDAC still have problems such as unsatisfactory safety. Although 5‐FU (5‐fluorouracil, 5‐pyridine), FOLFIRINOX (FOL‐folinic acid, F‐fluorouracil, IRIN‐irinotecan, OX‐oxaliplatin, folinic acid‐5‐fluorouracil‐oxaliplatin combined treatment regimen), gemcitabine and gemcitabine‐based adjunctive therapies have showed some effect in prolonging the patient's survival and improving their survival condition, the effect is not satisfactory. In recent years, immunotherapy has demonstrated outstanding effects in other types of malignant tumours, but its effect on PDAC patients is still poor. Therefore, it is a matter of great urgency to explore new treatment options based on previous experience.

In order to explore the key genes and potential targets in the development of PDAC, we performed a bioinformatics analysis and found that DIAPH3 in the formins family had a significantly high expression level in PDAC tissues than that in the para‐carcinoma tissues, and the high expression of DIAPH3 was significantly associated with a poor patient prognosis. In order to verify the above clinical findings, we carried out soft agar assay and confirmed that DIAPH3 could significantly promote the malignant phenotype of pancreatic cancer cells, and conducted a Transwell assay and showed that DIAPH3 can significantly promote the invasive capacity of pancreatic cancer cells, and also conducted MTS assay that showed DIAPH3 can significantly promote the proliferation ability of pancreatic cancer cells. The above results showed that DIAPH3 may be an important gene in the progression of pancreatic cancer. Previous studies reported that DIAPH3 promoted the growth, invasion and metastasis of liver cancer by activating β‐catenin/TCF signalling pathway.^23^ Interfering with DIAPH3 promoted tumour invasion and metastasis in mouse models.^22^ In addition, an overexpression of DIAPH3 inhibited the metastasis and invasive capacity of triple‐negative breast cancer by inhibiting the expression of RhoA‐GTP.^26^ Our results indicated that DIAPH3 promoted the malignant phenotype of pancreatic cancer cells. We analysed the interacting proteins of DIAPH3 in pancreatic cancer cell lines with mass spectrometry and performed a GO analysis. Results showed that the interacting proteins of DIAPH3 were mainly involved in the biological processes such as selenoamino acid metabolism, RNA splicing and ribonucleoprotein complex assembly. In contrast, the interacting proteins of DIAPH3 in other tumours, such as prostate cancer and glioblastoma, are tubulin and related microtubule dynamic regulating proteins. DIAPH3 promotes the stability of microtubules by interacting with microtubules. Therefore, we speculate that DIAPH3 may interact with different proteins in different tumours, thereby promoting or inhibiting tumour progression by different molecular mechanism. Therefore, we should adopt different DIAPH3‐targeting strategies to treat diseases according to its function and molecular mechanism of DIAPH3 in different tumours.

By using mass spectrometry, we detected the interaction of DIAPH3 with key proteins related to various selenoamino acid metabolism pathways in pancreatic cancer cell lines, such as RPS8, IARS, RPL4, RPL6, and RPS2, which suggested that we can inhibit the progression of pancreatic cancer by regulating the metabolism of selenoamino acids. Based on the bioinformatic analysis, proteins interacted with DIAPH3 identified with Mass spectrometry may be involved in selennoamino acid metabolism and we confirmed RPL6 was the only validated protein interacted with DIAPH3. Previous report indicates a significant functional cluster of ribosomal proteins including RPL6 was involved in selenium and selenoprotein levels maintenance.^31^ Specifically, deficiency in these ribosomal proteins alters the ribosome structure facilitating an increased Sec insertion. It is also possible that these knock downs decreased the rate of protein synthesis, which indirectly supported the inherently slow Sec insertion. Thus, the previous data point to potential control of selenoprotein expression through ribosome structure and function. Based on this paper and our observations, we infer that DIAPH3‐RPL6 interaction may alter the ribosome structure and increase Sec insertion and consequent selenoprotein TrxR1 expression. It is also possible that DIAPH3‐RPL6 interaction decreased the rate of protein synthesis, which indirectly supported the inherently slow Sec insertion and consequent selenoprotein TrxR1 expression. However, the specific mechanism of DIAPH3‐RPL6 interaction on TrxR1 expression requires further studies. Moreover, recent observations indicated breast and other cancer cells uptake selenium and promote selenocysteine biosynthesis, which, by allowing production of selenoproteins such as GPX4, protects cells against ferroptosis.^32^ Thus, selenium metabolism could promote malignancies of cancer cells via regulation of selenoproteins. We speculate DIAPH3 may promote selenoprotein (such as TrxR1 or GPX4) synthesis, which direct reduction of hydroperoxides or resist ferroptosis, respectively. Interestingly, we validated in vitro that DIAPH3 could promotes PDAC cells malignant phenotype via TrxR1‐mediated ROS levels and DIAPH3 knock down could decrease tumour growth and TrxR1 expression in vivo. Thus, our preliminary investigation demonstrates DIAPH3 at least partially promotes PDAC progression via TrxR1‐mediated ROS scavenging and TrxR1 may be a promising target for improved target therapy of PDAC, which is worthy of further investigation.

## CONFLICT OF INTEREST

The authors declare no conflict of interest.

## AUTHORS’ CONTRIBUTION

yefei rong: Data curation (equal); Formal analysis (equal). Jie Gao: Investigation (equal). Tiantao Kuang: Data curation (equal); Formal analysis (equal). Jian‐ang Li: Methodology (equal); Resources (equal); Software (equal). Jianlin Chen: Investigation (equal); Resources (equal); Software (equal). Yufeng Huang: Resources (equal); Software (equal); Supervision (equal). Haiguang Xin: Investigation (equal); Methodology (equal); Project administration (equal); Validation (equal). Yuan Fang: Investigation (equal); Resources (equal); Supervision (equal). Xu Han: Investigation (equal); Methodology (equal); Software (equal); Supervision (equal); Visualization (equal). Lunquan Sun: Software (equal); Supervision (equal); Validation (equal); Visualization (equal). Yue‐Zhen Deng: Supervision (equal); Writing‐original draft (equal); Writing‐review & editing (equal). Zhi Li: Conceptualization (equal); Data curation (equal); Formal analysis (equal); Supervision (equal); Validation (equal); Visualization (equal); Writing‐original draft (equal); Writing‐review & editing (equal). Wenhui Lou: Conceptualization (equal); Funding acquisition (lead); Supervision (equal); Visualization (equal); Writing‐review & editing (equal).

## Supporting information

Fig S1

Fig S2

Fig S3

## Data Availability

The data that support the findings of this study are available from the corresponding author upon reasonable request.

## References

[1] Rahib L , Smith BD , Aizenberg R , Rosenzweig AB , Fleshman JM , Matrisian LM . Projecting cancer incidence and deaths to 2030: the unexpected burden of thyroid, liver, and pancreas cancers in the United States. Cancer Res. 2014;74:2913‐2921. 24840647 10.1158/0008-5472.CAN-14-0155

[2] Burris HA 3rd , Moore MJ , Andersen J , et al. Improvements in survival and clinical benefit with gemcitabine as first‐line therapy for patients with advanced pancreas cancer: A randomized trial. J Clin Oncol. 1997;15:2403‐2413. 9196156 10.1200/JCO.1997.15.6.2403

[3] Hidalgo M . Pancreatic cancer. N Engl J Med. 2010;362:1605‐1617. 20427809 10.1056/NEJMra0901557

[4] Von Hoff DD , Ervin T , Arena FP , et al. Increased survival in pancreatic cancer with nab‐paclitaxel plus gemcitabine. N Engl J Med. 2013;369:1691‐1703. 24131140 10.1056/NEJMoa1304369 PMC4631139

[5] Moore MJ , Goldstein D , Hamm J , et al. National Cancer Institute of Canada Clinical Trials G. Erlotinib plus gemcitabine compared with gemcitabine alone in patients with advanced pancreatic cancer: A phase III trial of the National Cancer Institute of Canada Clinical Trials Group. J Clin Oncol. 2007;25:1960‐1966. 17452677 10.1200/JCO.2006.07.9525

[6] Garrido‐Laguna I , Hidalgo M . Pancreatic cancer: from state‐of‐the‐art treatments to promising novel therapies. Nat Rev Clin Oncol. 2015;12:319‐334. 25824606 10.1038/nrclinonc.2015.53

[7] Maitra A , Hruban RH . Pancreatic cancer. Annu Rev Pathol. 2008;3:157‐188. 18039136 10.1146/annurev.pathmechdis.3.121806.154305 PMC2666336

[8] Aguirre AJ , Nowak JA , Camarda ND , et al. Real‐time genomic characterization of advanced pancreatic cancer to enable precision medicine. Cancer Discov. 2018;8:1096‐1111. 29903880 10.1158/2159-8290.CD-18-0275 PMC6192263

[9] Bogdan S , Schultz J , Grosshans J . Formin' cellular structures: Physiological roles of Diaphanous (Dia) in actin dynamics. Commun Integr Biol. 2013;6:e27634. 24719676 10.4161/cib.27634 PMC3977921

[10] Sagot I , Klee SK , Pellman D . Yeast formins regulate cell polarity by controlling the assembly of actin cables. Nat Cell Biol. 2002;4:42‐50. 11740491 10.1038/ncb719

[11] Tominaga T , Sahai E , Chardin P , McCormick F , Courtneidge SA , Alberts AS . Diaphanous‐related formins bridge Rho GTPase and Src tyrosine kinase signaling. Mol Cell. 2000;5:13‐25. 10678165 10.1016/s1097-2765(00)80399-8

[12] Watanabe S , Ando Y , Yasuda S , et al. mDia2 induces the actin scaffold for the contractile ring and stabilizes its position during cytokinesis in NIH 3T3 cells. Mol Biol Cell. 2008;19:2328‐2338. 18287523 10.1091/mbc.E07-10-1086 PMC2366861

[13] Castrillon DH , Gonczy P , Alexander S , et al. Toward a molecular genetic analysis of spermatogenesis in Drosophila melanogaster: characterization of male‐sterile mutants generated by single P element mutagenesis. Genetics. 1993;135:489‐505. 8244010 10.1093/genetics/135.2.489 PMC1205651

[14] Alberts AS . Identification of a carboxyl‐terminal diaphanous‐related formin homology protein autoregulatory domain. J Biol Chem. 2001;276:2824‐2830. 11035012 10.1074/jbc.M006205200

[15] Castrillon DH , Wasserman SA . Diaphanous is required for cytokinesis in Drosophila and shares domains of similarity with the products of the limb deformity gene. Development. 1994;120:3367‐3377. 7821209 10.1242/dev.120.12.3367

[16] Shinohara R , Thumkeo D , Kamijo H , et al. A role for mDia, a Rho‐regulated actin nucleator, in tangential migration of interneuron precursors. Nat Neurosci. 2012;15(373–80):S1‐2. 10.1038/nn.3020 22246438

[17] Watanabe S , Okawa K , Miki T , et al. Rho and anillin‐dependent control of mDia2 localization and function in cytokinesis. Mol Biol Cell. 2010;21:3193‐3204. 20660154 10.1091/mbc.E10-04-0324 PMC2938385

[18] Schoen CJ , Emery SB , Thorne MC , et al. Increased activity of Diaphanous homolog 3 (DIAPH3)/diaphanous causes hearing defects in humans with auditory neuropathy and in Drosophila. Proc Natl Acad Sci U S A. 2010;107:13396‐13401. 20624953 10.1073/pnas.1003027107 PMC2922180

[19] Bartolini F , Moseley JB , Schmoranzer J , Cassimeris L , Goode BL , Gundersen GG . The formin mDia2 stabilizes microtubules independently of its actin nucleation activity. J Cell Biol. 2008;181:523‐536. 18458159 10.1083/jcb.200709029 PMC2364705

[20] Bartolini F , Ramalingam N , Gundersen GG . Actin‐capping protein promotes microtubule stability by antagonizing the actin activity of mDia1. Mol Biol Cell. 2012;23:4032‐4040. 22918941 10.1091/mbc.E12-05-0338 PMC3469518

[21] Wen Y , Eng CH , Schmoranzer J , et al. EB1 and APC bind to mDia to stabilize microtubules downstream of Rho and promote cell migration. Nat Cell Biol. 2004;6:820‐830. 15311282 10.1038/ncb1160

[22] Reis‐Sobreiro M , Chen JF , Novitskaya T , et al. Emerin deregulation links nuclear shape instability to metastatic potential. Cancer Res. 2018;78:6086‐6097. 30154147 10.1158/0008-5472.CAN-18-0608

[23] Dong L , Li Z , Xue L , et al. DIAPH3 promoted the growth, migration and metastasis of hepatocellular carcinoma cells by activating beta‐catenin/TCF signaling. Mol Cell Biochem. 2018;438:183‐190. 28795316 10.1007/s11010-017-3125-7

[24] Hager MH , Morley S , Bielenberg DR , et al. DIAPH3 governs the cellular transition to the amoeboid tumour phenotype. EMBO Mol Med. 2012;4:743‐760. 22593025 10.1002/emmm.201200242 PMC3494074

[25] Stastna J , Pan X , Wang H , et al. Differing and isoform‐specific roles for the formin DIAPH3 in plasma membrane blebbing and filopodia formation. Cell Res. 2012;22:728‐745. 22184005 10.1038/cr.2011.202 PMC3317560

[26] Jiang J . Diaphanous‐related formin‐3 overexpression inhibits the migration and invasion of triple‐negative breast cancer by inhibiting RhoA‐GTP expression. Biomed Pharmacother. 2017;94:439‐445. 28779705 10.1016/j.biopha.2017.07.119

[27] Kong H , Chandel NS . Regulation of redox balance in cancer and T cells. J Biol Chem. 2018;293:7499‐7507. 29282291 10.1074/jbc.TM117.000257 PMC5961053

[28] Busker S , Qian W , Haraldsson M , et al. Irreversible TrxR1 inhibitors block STAT3 activity and induce cancer cell death. Sci Adv. 2020;6:eaax7945. 32219156 10.1126/sciadv.aax7945 PMC7083616

[29] Liou GY , Storz P . Detecting reactive oxygen species by immunohistochemistry. Methods Mol Biol. 2015;1292:97‐104. 25804750 10.1007/978-1-4939-2522-3_7 PMC4571457

[30] Adamska A , Domenichini A , Falasca M , Falasca M . Pancreatic ductal adenocarcinoma: Current and evolving therapies. Int J Mol Sci. 2017;18(7):1338. 10.3390/ijms18071338 PMC5535831 28640192

[31] Malinouski M , Hasan NM , Zhang Y , et al. Genome‐wide RNAi ionomics screen reveals new genes and regulation of human trace element metabolism. Nat Commun. 2014;5:3301. 24522796 10.1038/ncomms4301 PMC5578452

[32] Carlisle AE , Lee N , Matthew‐Onabanjo AN , et al. Selenium detoxification is required for cancer‐cell survival. Nat Metab. 2020;2:603‐611. 32694795 10.1038/s42255-020-0224-7 PMC7455022

