# Supplementary figures and images for: DIAPH3 promotes pancreatic cancer progression by activating selenoprotein TrxR1‐mediated antioxidant effects
Source: J Cell Mol Med. 2020 Dec 20;25(4):2163–75. doi: 10.1111/jcmm.16196 (PMC7882936; doi:10.1111/jcmm.16196)

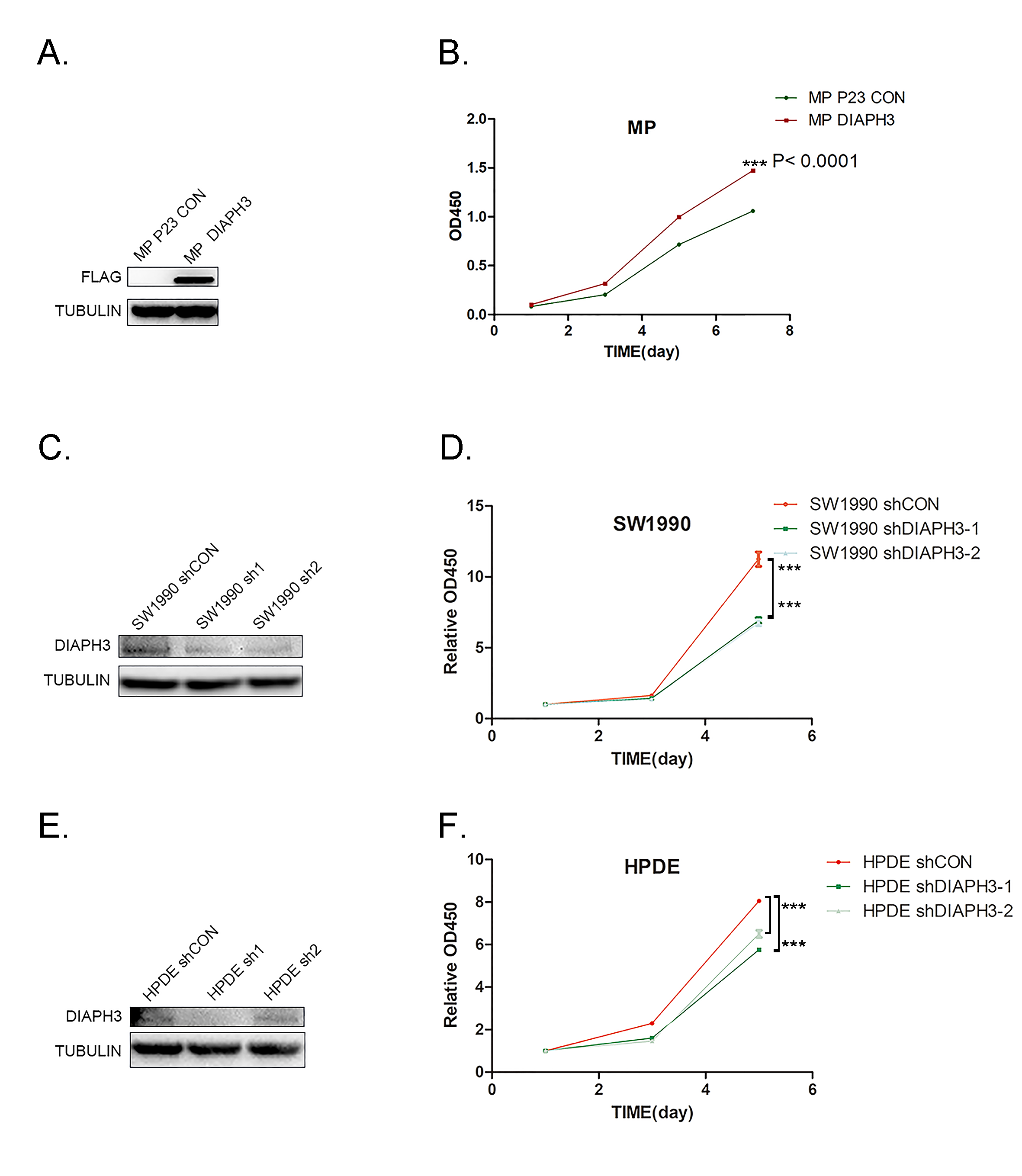

Supplement: Supplementary file 1 — Fig S1 [file JCMM-25-2163-s001.tif]

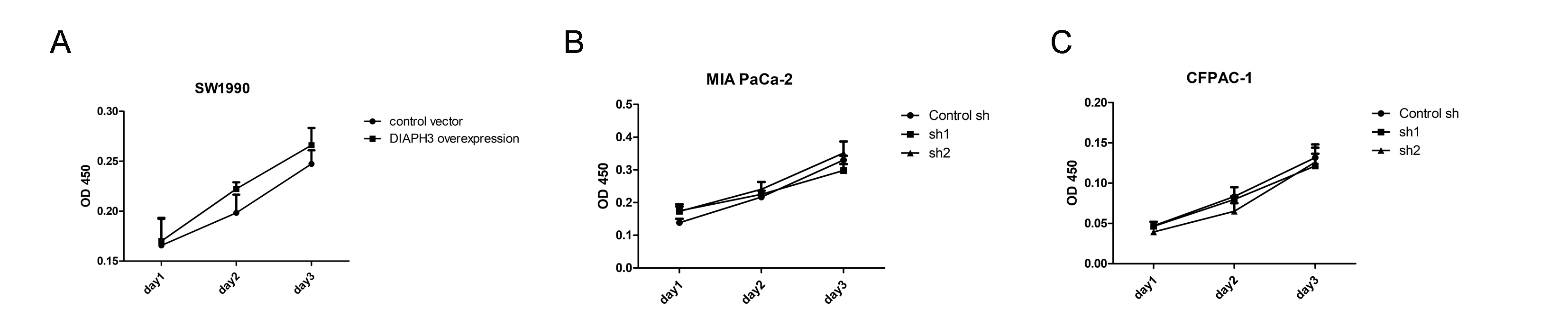

Supplement: Supplementary file 2 — Fig S2 [file JCMM-25-2163-s002.tif]

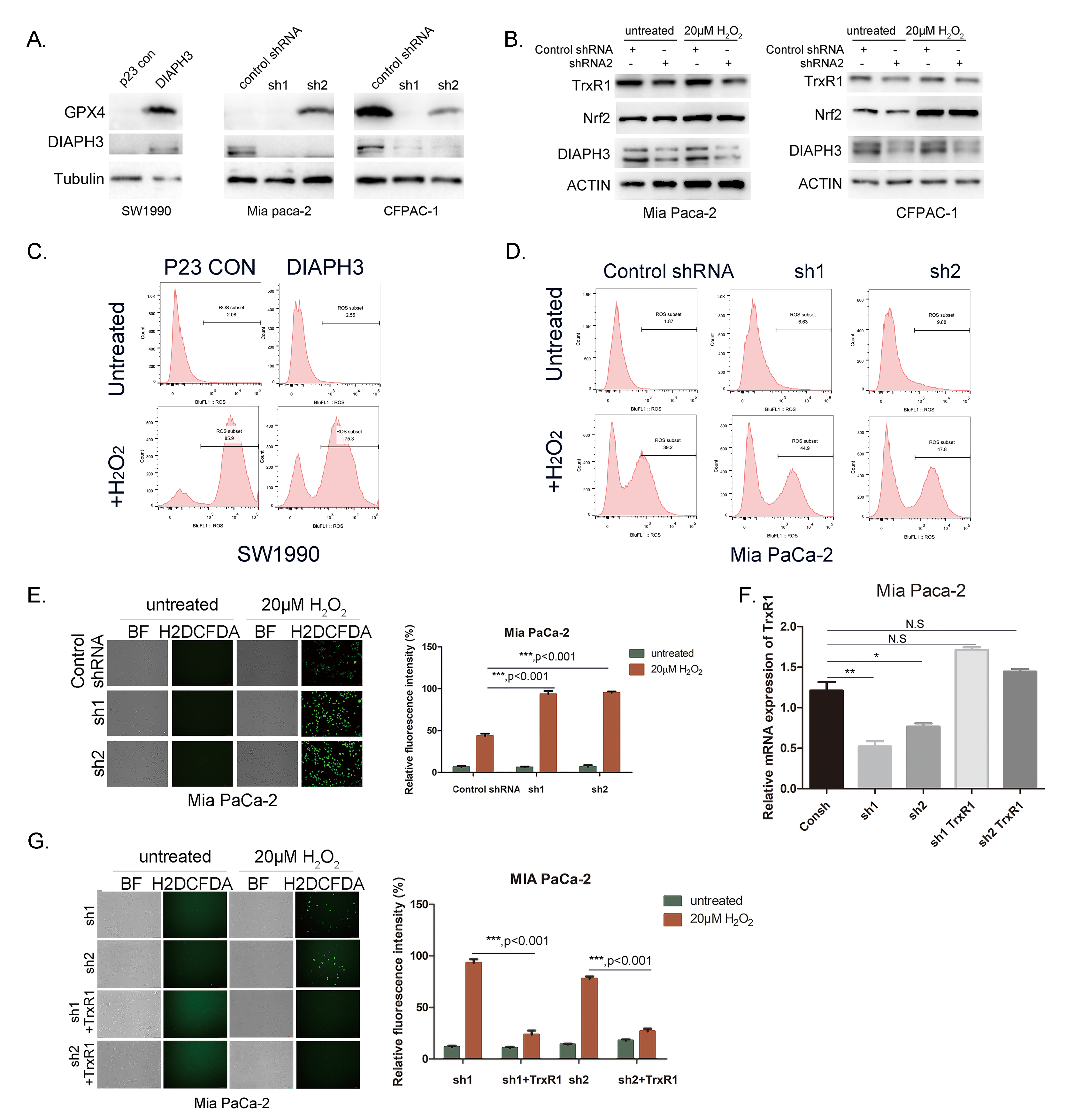

Supplement: Supplementary file 3 — Fig S3 [file JCMM-25-2163-s003.tif]
